# Improving Social Interactions Among Adult Carers of Children with Disabilities Through Dialogic Literary Gatherings: A Case Study from Pakistan

**DOI:** 10.3390/bs15040509

**Published:** 2025-04-10

**Authors:** Shiza Khaqan, Gisela Redondo-Sama, Ramon Flecha

**Affiliations:** 1 Department of Pedagogy, University Rovira i Virgili, 43003 Tarragona, Spain; gisela.redondo@urv.cat; 2 Department of Sociology, University of Barcelona, 08007 Barcelona, Spain; ramon.flecha@ub.edu

**Keywords:** social interactions, dialogic literary gatherings, well-being, South Asia, self-development

## Abstract

The scientific literature has evidenced that certain educational and dialogic actions have been successful at increasing the quality of social interactions and improving mental health, among other beneficial aspects. One of these dialogic actions is the dialogic literary gathering (DLG). The impacts of DLGs have been demonstrated in Europe and South America but have yet to be explored in Eastern culture. The goal of the current study was to analyze the transferability of DLGs within South Asian culture in Pakistan. Specifically, the aim was to identify how the DLGs impacted the social interactions of middle-aged to older adults who were caregivers for children with disabilities. This research used a qualitative case study design with a communicative methodology to study a group of adult family members at a special education school who participated in the DLGs. Data collection included interviews, focus groups, and observations, and a thematic analysis was carried out. Based on the analysis, four broad categories emerged: cognitive engagement, mental well-being, self-development, and social–emotional experiences. Overall, the results demonstrate that the DLGs facilitated positive self-changes among participants, enabling them to experience and express a wide range of emotions through social interactions.

## 1. Introduction

The current dialogic society (Flecha, 2022) enables social interactions based on egalitarian principles and contributes to the creation, development, and sustainability of practices that overcome loneliness as well as social isolation, including among vulnerable populations such as adults in challenging contexts. Dialogue that takes place during social interactions holds great power for developing solutions, forming opinions, and bringing people closer (Ellinor & Girard, 2023). This study focused on the significance of dialogue during social interactions among middle-aged to older adults during the implementation of a dialogue-based intervention aimed at increasing social interactions in Pakistan: dialogic literary gatherings (DLGs). These adults are the caregivers of children with disabilities who generally have additional caregiving and financial responsibilities, as compared with other parents. The following sections review the literature on social interactions, including their development and scarcity, and parents within the disability context in Pakistan and provide a brief overview of DLGs.

### 1.1. Social Interactions and Loneliness During Adulthood

Social interactions lay at the foundation of most rewarding outcomes such as finding a job or a romantic partner and building strong relationships (Krach et al., 2010). Therefore, they play an important role throughout a lifespan. During young to middle adulthood, individuals normally make many social connections. However, in certain situations, social interactions may decline and individuals may be at risk of isolation. One such situation occurs in later adulthood, around the 60s, when individuals begin to experience a decline in their socialization and loss of meaningful relations, leading to feelings of loneliness (Lutfey & Mortimer, 2003). Here, it is crucial to distinguish between social isolation and loneliness. While social isolation is an objective state, defined by a lack of social interactions and participation in social activities, loneliness is a subjective experience characterized by the perception of being alone, which may or may not stem from actual social isolation (Holt-Lunstad et al., 2015). For example, while some individuals living in social isolation may find contentment in their solitude, those experiencing loneliness typically perceive it as an undesirable state (Holt-Lunstad et al., 2015). As a result, loneliness is often associated with negative psychological and emotional outcomes and is increasingly being recognized as a significant public health concern (Cacioppo & Cacioppo, 2018). A gradual decline in social interactions may occur unintentionally due to retirement, relocating after retirement, or losing close family and friends to illness. Loneliness brought about by such social isolation can have numerous adverse consequences, such as depression and anxiety disorders, suicide, health complications such as coronary disease, and premature mortality (Donovan & Blazer, 2020).

Chances of social isolation are exacerbated in individuals from at-risk or vulnerable groups, including parents of children with a chronic illness or disability (Nowland et al., 2021). The demands of long-term caregiving can lead to increased stress levels, which have been associated with accelerated cognitive decline, particularly in episodic memory among older mothers (Seltzer et al., 2011). Furthermore, the caregiving role often requires personal sacrifices, including reduced social interactions and postponed personal needs, which can impact the caregiver’s emotional well-being and quality of life (Roger et al., 2000). The transition to parenthood itself can be a reason for decrease in social interactions among parents due to the change in routines and activities; however, other reasons can also be regret associated with having a child and the social stigma in case of a child with disabilities (Duran & Ergün, 2018) or social restrictions such as those during the pandemic (Bodin & Fängström, 2025). Parents experiencing loneliness due to such factors are often socially isolated due to childcare responsibilities and long for adult company (Bodin & Fängström, 2025). For parents of children with disabilities, social isolation and consequential loneliness is greater, which may be attributed to difficulty in socializing with other friends and families due to the physical or social challenges that their child may struggle with (George-Levi et al., 2024), along with the existing social stigma surrounding children with disabilities (Duran & Ergün, 2018). The social context for the parents is moderated by the cultural context they live in. In Western countries, parents and older adults benefit more from interventions that set personal growth goals, whereas in collectivist cultures such as in South Asia, they benefit more from interventions that involve family or community and set collective goals (Hartanto et al., 2024). Overall, individuals in middle and later adulthood, as well as parents of children with disabilities, are more likely to experience loneliness due to social isolation. Consequently, parents who fall into both categories are understandably at an even greater risk of facing a lack of social interactions. Despite these challenges, some caregivers find fulfillment in their roles, reporting a sense of purpose and positive emotional experiences, especially when they have strong social support networks (Greenfield & Marks, 2004). Nonetheless, the cumulative stress associated with caregiving responsibilities highlights the need for targeted support and interventions to assist aging caregivers in maintaining their health and well-being. 

### 1.2. Experiences of Parents of Children with Disabilities in Pakistan

According to a UNICEF report, in Pakistan, among children aged 8 to 12 years old, 11 percent have some type of disability (Grimes, 2021). In Pakistan, with little to no formal support for parents and caregivers of children with disabilities, they rely mainly on family and friends for help (Lakhani et al., 2024). However, such help may not always be readily available and the parents of children with such conditions have significant levels of stress and caregiver burden (Syed et al., 2020). Specifically, mothers of children with intellectual disabilities face higher levels of depression and anxiety than fathers (Azeem et al., 2013). The underlying cause of this difference has been explained in terms of gender roles and responsibilities associated with motherhood, as in Pakistan, mothers are mostly responsible for the childcare; therefore, this is understood to be the reason for the higher levels for distress (Azeem et al., 2013).

Within Pakistani society, there is significant social stigma against disability which is a barrier for inclusive education (Kamran & Bano, 2023). Additionally, inadequate educational infrastructure and a shortage of trained teachers for children with disabilities further contribute to structural ableism (Kamran & Bano, 2023). Furthermore, the social stigma surrounding disability often subjects their children to social scorn, prompting some parents to avoid public outings and become more socially withdrawn (Rizvi Jafree & Burhan, 2020). Such social isolation can have adverse impacts on the parents’ perceived health (Thompson et al., 2020) and on parenting practices resulting in child maltreatment in the form of neglect or verbal aggression (Lee et al., 2022).

Considering the prevalence of poverty in Pakistan, most parents also report their children to be a financial challenge, a challenge that becomes considerably greater for children with disabilities (Ramzan et al., 2022), another factor highlighting systemic ableism. In addition, parents from lower socioeconomic backgrounds often experience marginalization in education, as the high costs of books, uniforms, special equipment, and other school supplies can be difficult to afford (Pirzada et al., 2024). The lack of community initiatives and community cohesion for children with disabilities and their families puts them at a further disadvantage when compared with children without disabilities (Kamran & Bano, 2023). Therefore, the parents and caregivers who raise children with disabilities in Pakistan and belong to a lower socioeconomic class face considerable challenges which threaten their well-being and put them at a higher risk of social isolation and marginalization.

### 1.3. Aging Caregivers of Children with Disabilities

Research also indicates how some parents of children with developmental disabilities view their caregiving experiences as an extension of their own self-actualization, as these carers find a sense of purpose and identity within their caregiving role (Avieli et al., 2022). However, not all parents entering old age view caregiving during old age as a means for self-actualization, as they report feeling worn out but persist out of necessity (Band-Winterstein & Avieli, 2017). So, some parents view caregiving as a mission or a full-time commitment and others find ways to balance personal and familial goals alongside their responsibilities (Avieli et al., 2022).

In Pakistan, where there is a growing population of older adults, expected to reach 43.3 million by 2050, cultural norms emphasize family-based elder care (Shekhani, 2024). Caregiving responsibilities are often placed on women, such as daughters-in-law, yet limited formal support services create challenges for such caregivers (Shekhani, 2024). In such situations, women caring for both aging family members and children with disabilities face compounded challenges as they enter later adulthood, balancing multiple caregiving responsibilities while managing their own aging-related concerns (Munir, 2016). Caregivers of children with disabilities in Pakistan present with high levels of caregiver burden, which may be attributed to the high level of expenses that need to be borne by the carers in contrast to government-provided care (Saeed et al., 2024). Older caregivers who already have no or a very limited source of income may find this responsibility increasingly distressful within such a context.

### 1.4. Dialogic Literary Gatherings—A Dialogic Intervention

Dialogic literary gatherings (DLGs) have demonstrated positive social- and well-being-related outcomes, with a focus on reading and discussing a literary classic as a group (Morlà-Folch et al., 2022). The first DLG was carried out in an adult education school in Barcelona in an impoverished neighborhood. Since the adults in this neighborhood had limited formal education, the goal of the DLG was to promote literacy and strengthen their relationships (Aubert et al., 2016). Prior research shows that engaging in DLGs over time leads to improved learning outcomes (García-Carrión & Díez-Palomar, 2015), enduring friendships (Serradell, 2015), increased social activism (Yeste et al., 2017), improved quality of life, and a reduction in loneliness (León-Jiménez, 2020). DLGs emphasize the equality of differences in dialogue, stressing the equal participation and inclusion of all participants and recognizing their right to live differently (Ruiz-Eugenio et al., 2023). It is this focus on engaging community members with diverse backgrounds and facilitating egalitarian dialogue that enables the achievement of the positive outcomes of DLGs (Padrós & Flecha, 2014). They have been especially instrumental in empowering historically marginalized groups such as the Roma (López de Aguileta, 2021), as it has enabled them to develop their leadership potential, called dialogic leadership (Redondo-Sama, 2015). Additionally, research indicated that participation in DLGs improved the familial relationships and the quality of life of the families participating (Elboj-Saso et al., 2021). DLGs have been implemented across Europe, South America, and Africa (Allotey et al., 2023; López de Aguileta, 2021). The impacts of this intervention have not yet been explored in South Asia.

### 1.5. Objective

The overarching aim of this study was to implement and analyse the impacts of DLGs on the social interactions of middle-aged to older adults who are caregivers for children with disabilities in Pakistan at a school. This school operates under a non-profit organization and provides low-cost education for children with disabilities. The adults at the core of the study are at the intersection of three vulnerable groups, low- to middle-income families, middle-aged to older adults, and carers of children with disabilities, all of whom face heightened risks of social isolation and loneliness. This particular case was chosen as it has a context similar to that of the adult education school where DLGs were initially conceived and developed. With members with similar educational and socioeconomic backgrounds and age groups, and with children with disabilities, the rationale behind carrying out the DLGs was to promote learning and social interactions, leading to improvements in well-being. The aim of this study was to describe and analyse the experiences of participating in the DLGs, contextualizing the findings to a South Asian context, and identifying how the DLGs impacted the social interactions of middle-aged to older adults who are caregivers for children with disabilities.

## 2. Materials and Methods

### 2.1. Design

The aim of the study was to find out about the experiences of the participants using a qualitative study with a communicative focus framed by a communicative methodology (CM), which ensured the inclusion of the voices of the participants as social actors in the research process (Gomez et al., 2013). CM includes dialogic practices that result in the co-creation of meaning from various diverse voices, inclusive of those within marginalized communities. As CM has been shown to be a useful method to assess social impact (Redondo-Sama et al., 2020), it was chosen for this study.

### 2.2. Recruitment and Participants

This study was carried out at a school in Lahore, Pakistan, which caters specially to children with intellectual and developmental disabilities from an underprivileged background. They offer a low tuition fee and have several volunteers, mainly university students, who help at the school in addition to the staff and teachers. The initial contact with the school was made by one of the researchers who works in the field of disabilities with a background in grassroots organizations, helping to frame this study according to its aims and potential of developing DLGs. When the purpose of the study was explained to the school principal and the staff members, they expressed what they wished to be incorporated in the study. They described the demographics and the situation of the potential participants in the DLGs, which helped the researchers to frame the study accordingly, keeping in view their age group, socioeconomic status, and roles as parents/family members of children with disabilities. The staff members in the school also stated that the book used in the DLGs should have relevance to the participants’ lives and that it should have a meaningful message. This dialogue between the researchers and the staff members to plan the DLGs framed this study and the potential analysis and results. The research team suggested literary classics accordingly, and in collaboration with the staff of the school, prepared a flyer containing information about the activity and the study, in non-academic and engaging language. The flyer was circulated among the family members, inviting them to participate. Initially, 12 family members agreed to participate; however, as 2 of them were not available for the data collection, the final number consisted of 10 participants. They were middle-aged to older adults, with an average age of 51.7 years and with an age range of 38 to 64. Among them, 7 were women and 3 were men. All of the participants were parents of children with different levels of intellectual and developmental disabilities and from ages of 3 to 14, except for one woman, who was a grandparent.

### 2.3. Materials

The materials developed for this study included the ethical procedure materials, guides for interviews and focus groups, and observation sheets. Regarding the ethical procedure materials, an informed consent form and a demographics questionnaire were used to provide participants with information about the research, obtain their consent, and record their demographic details, respectively. These materials were developed in accordance with the guidelines, procedures, and approval of the Ethics Committee of the university. With regard to the guides for the interviews and focus groups, they were developed by the research team according to the aim of this study and co-creation processes. The guides included between 10 and 12 initial questions that were followed up on and narrowed down during the interviews and the focus groups, integrating a transformative dimension used in CM (Molina Roldán, 2015).

Further, an observation sheet was used to record the researchers’ observations after each session. This observation sheet was developed collaboratively among the researchers, taking into account that it has been used in previous investigations to record observations during other dialogue-based interventions (Morlà-Folch et al., 2020). The sheet had 4 items measuring observations about learning, engagement, and participation on a 5-point Likert scale ranging from ‘Strongly agree’ to ‘Strongly disagree’. Participation was defined in terms of the frequency of verbal contributions made during the sessions, while engagement was conceived as a deeper level of involvement in the sessions, characterized by body language cues such as eye contact and hand gestures in addition to active listening and meaningful contributions. Additionally, the observation sheet contained four open-ended items related to the themes discussed during the DLGs, the type of engagement, and any other noteworthy observations.

### 2.4. Procedure

Initially, the participants were informed by the staff of the school about the DLGs and they were asked to choose the literary classic they would like to read and the day/s and the duration of the session they would like to meet for, keeping in line with the democratic principles of DLGs (López de Aguileta et al., 2020). From the options given, they chose the Urdu literary classic ‘Patras kay Mazameen’ published in 1929, which is a collection of essays with a satirical perspective of social and political life in the sub-continent in the early 1900s (Wikipedia Contributors, 2023). The participants were provided with a free copy of the book, with a stationary set for each participant. The participants also decided to meet once every week for a one-and-a-half-hour-long session.

The DLGs started with a welcome session by the researcher acting as a moderator, in which the procedure of the DLGs and the prompts underpinning their development were explained in depth to the participants. The researcher shared that the discussion would be opened by asking the participants about who would like to speak about a certain part of the reading. Following this, the researcher would ask the first person, “Would you like to share the part you wanted to highlight?”. After this prompt, the researcher would ask, “Would anyone like to comment on this?”, and then, the participants could continue with the dialogues about the selected part of the book.

Before the DLG sessions, the participants were informed that they should choose a part of the book that they would like to read and discuss during the session. They marked any part that they found meaningful so that they could highlight and speak about it. During the session, the researcher acting as the moderator noted down the names of all those who would like to speak about a certain part. Then, after they spoke, the other participants also commented and discussed their chosen passage. In this way, the participants spoke about many topics related to their life, their children, Pakistani society and culture, and challenges they face while raising a child with disabilities.

The DLGs lasted for two months and an attendance record was maintained while the participants and researcher remained in direct contact so that the participants would inform them beforehand if they were unable to attend a session. All participants were present in all the sessions, except for 3 sessions in which either 2 or 3 participants were not able to attend for major reasons. Once the DLG sessions were finalized, the data collection began and each participant was interviewed. Each participant also participated in either one of the two focus groups that were conducted. Each interview lasted for about twenty minutes to half an hour and each focus group lasted for about 30 to 45 min. A semi-structured approach to the interviews was taken. The purpose of the interviews and the focus groups was to find out and explore in-depth the perceived impacts that the participants perceived the DLGs to have had on them at an individual level. Data were also collected in the two focus groups in which participants shared their experiences related to the DLGs. Further, the researcher recorded observations after each session to note the level of engagement and participation, using the observation sheet. No reimbursement or incentive was given to the participants for their participation in the DLGs and subsequent data collection.

### 2.5. Analysis

Before the analysis, the interviews which were conducted in Urdu were transcribed with Whisper AI, which has demonstrated high accuracy in processing conversational Urdu (Arif et al., 2024). They were then translated into English for the analysis using DeepL, due to its advanced contextual understanding (Telaumbanua et al., 2024). To ensure reliability, the translated text was reviewed by the researcher, a native Urdu speaker with high proficiency in English, confirming that the vocabulary, phrasing, and overall coherence remained accurate and consistent. A few minor translation errors were corrected to ensure that the transcripts remained true to the original meaning. Further, back-translation was used with a different software tool, Google Translate, to confirm that the translation was accurate.

After this, the data were analyzed using Braun and Clarke’s approach to thematic analysis (Braun & Clarke, 2006). The software ATLAS.ti 24 was used to store and organize the data and to assist in coding. In accordance with the framework, the first step carried out was familiarization with the data. During this stage, the interview and focus group transcripts were collaboratively read in depth by two members of the research team to gain a deep understanding of the participants’ experiences. Secondly, the process of initial code generation was initiated by manually assigning meaningful codes using the open coding function in ATLAS.ti. After this, the AI-assisted coding feature in ATLAS.ti was utilized to generate additional codes. This allowed the researchers to compare and contrast the manually created codes with those suggested by the AI, ensuring that no significant patterns or insights were overlooked. Recognizing the importance of human oversight, open coding with AI-assisted coding was combined to capture a full range of codes while minimizing potential biases that may arise from using only AI-assisted coding (Christou, 2024).

The AI-generated codes differed from the researcher-generated codes primarily in their breadth and quantity. The AI coding produced a large number of codes, capturing a wide range of patterns in the data. In contrast, the manual coding was more intentional and guided by the specific objectives of this study. However, there was significant overlap between the two approaches. In some cases, the AI identified additional codes that were identified as useful and incorporated into the analysis while the primary analysis was conducted through intentional, researcher-led coding, which inherently reflected the aim of the study. This stepwise process ensured that the interpretive lens remained central to the analysis, while the AI served as a complementary tool to enhance the human understanding of the data.

In the next step, “Searching for themes”, two members of the research team manually and collaboratively reviewed all the codes generated in the previous step and grouped similar codes into broader themes. The networks generated by ATLAS.ti were also examined, comparing them to the manually developed themes. While there was substantial alignment, the themes were finalized based on the specific context of this study. In the “Review of Themes” step, two members of the research team carefully examined the developed themes and their associated codes. Both researchers independently reviewed the themes and shared them, making minor adjustments until reaching a consensus that the themes aligned with the research aim. In the final two steps, “Defining themes” and “Writing the report”, the names of the finalized themes were refined to ensure that they accurately addressed the research aim and captured the essence of the data. Then, all members of the research team shared the results of the steps followed and proceeded with writing the report, synthesizing the findings and analysis. Table 1 presents the themes and subthemes of this study.

### 2.6. Reflexivity Statement

The researchers acknowledge that their diverse backgrounds, research, and work experiences influenced the research process. The research team has experience in working with vulnerable populations, with backgrounds in studying child and family psychology, working with children with disabilities and their families from a lower socioeconomic class in developing countries, families of children without disabilities from lower socioeconomic and migrant backgrounds, the Roma population, and children subjected to bullying. The research with these vulnerable groups may have heightened the sensitivity of the researchers to their challenges and, in particular, to the problems faced by the parents. The researchers remained cognizant about this and remained in reflexive dialogue about it, aligning the research with the egalitarian dialogue among the researchers and the people involved in the research process, as developed in CM (Gomez et al., 2013).

### 2.7. Ethical Aspect

This study was conducted in compliance with the 1975 Declaration of Helsinki and received ethical approval from CEIPSA, the ethics committee of University Rovira i Virgili (Ref no: CEIPSA-2023-TD-0051). To ensure the anonymity of the participants, they were given a code, and in all data collection documents, they were identified only by these codes. Further, to ensure confidentiality, the data were saved only on the researcher’s laptop, to which no one else had access. At the commencement of this study, all participants were clearly informed about the purpose of the study and of the voluntary nature of their participation with the informed consent sheet which included the right to withdraw at any time in the study. For the analysis, the primary focus was on researcher-led coding, ensuring depth and contextual understanding, while AI-assisted coding in ATLAS.ti was used only as a supplementary tool to support the analysis, with human interpretation remaining central to the process.

## 3. Results and Discussion

This study focused on carrying out and analyzing DLGs with Pakistani adults who were middle-aged to older carers (parents and grandparents) of children with disabilities. As a result of the analysis, four themes were identified: cognitive engagement, mental well-being, self-development, and social–emotional experiences. Each of these themes are described and discussed in the following sections.

### 3.1. Cognitive Engagement

During the DLG sessions, the social interactions among the adult carers in which they related the content of the book to their lives, comparing and critiquing the ideas, led them to engage mentally. During one of the sessions, a father brought up a verse that was written in the chapter of the book they had read. Although the book was in Urdu, the verse was in Persian and not understood by all. As he had studied Persian, he shared its meaning and his interpretation of it, and others also offered their opinions. In relation to this, one of the parents had said,

“When we read books and then discuss their content with other people, there is a lot of value to this activity. I think that the content got clearer when we talked about it.”

(P1)

Similarly, during other instances, participants shared new vocabulary that they learnt or different usage of words they already knew during the sessions. As two of the participants had never had any formal education, it was the first time that they were reading a literary classic. So, engaging with complex language and interpreting nuanced themes was new for them. Overall, these instances reflected the learning aspect of the DLGs, in which participants gained new knowledge from the sessions. This is consistent with previous findings which found that as a result of DLGs, learning was strengthened when used as an intervention to aid in schoolwork (Aguileta, 2019) and in language learning (Santiago-Garabieta et al., 2023). The reasons behind why DLGs have been seen as successful at improving dialogue may be to do with the format of the activity. For example, participants talk freely and critically examine and contribute to the discussion in a group format. This is similar to cooperative learning which has been demonstrated to have better learning outcomes than traditional lectures (Yamarik, 2007). As in DLGs, there is more comprehension of the topic when participants sit in a group and discuss, asking questions and making comments.

Similarly, the discourse seemed to compel the participants to think critically while conversing, especially when they were standing for or against an opinion. The text made them engage in a critical comparison of cultural and social practices. Relating it to their lives, they critiqued the stigma surrounding disabilities in Pakistani culture, which they often face in situations involving their children. They discussed ways in which policies should be altered to accommodate the individuals and their care takers in a better way. One of the mothers described this aspect of the gatherings in the following words:

“This book is quite old and most of the essays are based in Lahore, so it was good to compare what is happening now to what was happening then. I liked the aspect of talking critically about how the social life changed and whether it was for the better or worse. We had a bit of a debate (laughs), and this was an unusual way of getting to know each other’s opinions.”

(P9)

In this quotation, the mother is making a reference to a certain conversation in which opinions were quite divided on the change within social life over the years. It was notable that the participants were quite vocal about their opinions. For example, a father who would not initially speak to the women (due to religious and traditional norms) addressed a mother and asked her to explain her opinion. So, the activity helped to stimulate their cognitive engagement, as research indicates that engaging with classical literature is conducive to critical thinking (Levin, 2023). Previously, it has been demonstrated that participation in DLGs leads to the development of creative thinking among older adults (Aubert et al., 2016), and the current findings provide further evidence for this.

In addition to these mental skills, another cognitive skill that was utilized was perspective-taking. With the emphasis on the dialogue being egalitarian and everyone taking turns to speak, the participants listened to the comments by the others to comment on them. This made them consider their point of view, which they may not have considered before. For example, the mothers often spoke about household responsibilities and taking care of older family members and how challenging everything can be while caring for a child with disabilities. As most of the men and women had traditional gender roles where men went to work and took care of tasks outside the home, the women assumed the responsibilities of household tasks. So, during these discussions, the men heard and considered the women’s perspective and vice versa. In the words of a father, this sentiment was expressed in the following way:

“I think that we are only seeing one aspect of life, through our own eyes and our own problems. However, in each of these sessions, I was able to see a different aspect of life, through the lens of other peoples’ perspectives.”

(P11)

Overall, the DLGs increased the cognitive engagement among the participants, which aligns with previous research identifying that participation in DLGs contributes to mental engagement, specifically learning (Aubert et al., 2016; García-Carrión et al., 2020). Specifically, DLGs have shown to support mental health outcomes by improving mentalizing, the ability to understand mental states (Fernández-Villardón et al., 2025). One of the aspects of mentalizing is perspective-taking, whose development during the DLGs this study also provides further evidence for.

Dialogue plays an important role in developing cognitive skills such as problem solving and reasoning, as speaking to another person helps to think more coherently and to arrive at a solution (Mercer & Howe, 2012). This cognitive aspect of this intervention was highly significant for this group of aging adults as many parents entering old age start to report signs of cognitive decline such as failing to remember certain episodes (Seltzer et al., 2011). Additionally, other dialogue-based interventions have shown to improve reasoning, higher-order thinking, and argumentation (García-Carrión & Díez-Palomar, 2015), which further strengthens the role of dialogue in enabling cognitive engagement.

### 3.2. Mental Well-Being

In their experiences of attending the DLGs, the participants expressed how they experienced a reduction in their stress levels after attending the sessions. As the participants belonged to lower- to middle-income backgrounds, with other children and often older family members to care for, they had busy lives, often with little to no time for themselves. It is common for parents of children with disabilities to experience parental burnout and stress (Findling et al., 2023). In the present study, the parents chose such a time for the DLG sessions so that it would coincide with when the children finished their lessons, and the session would merge into their routine. Participants expressed their sentiments about the sessions in the following words:

“For me and my wife these gatherings were very useful. We are normally quite stressed most days, and I think the other parents too would be, as most parents of children with disabilities remain stressed. So, participating in the DLGs helped a lot in relieving this stress.”

(P4)

“I think it was a good way for us to overcome the many worries in our lives. It’s not just us but most of the people in our society are dealing with many problems such as financial constraints etc. I think if more people take part in such sessions, they will benefit from them.”

(P4)

In addition to experiencing a sense of relief, the opportunity to speak freely without judgement was also something unique and that provided them with a source of catharsis.

“We felt that we could share anything here like in our family. It’s like how you feel lighter after talking to a friend. That’s what I enjoyed.”

(P6)

The quotations reflect the kind of atmosphere the participants perceived, which was friendly and non-judgmental, where their opinions were listened to and valued. This can be understood in psychological terms as a form of therapy, talk therapy, where one can speak candidly with a professional, which has been proven to cause an improvement in mental well-being (Fenger-Grøn et al., 2018). Also, sharing life experiences with other people has been associated with improvements in certain aspects of mental well-being like empathy (Cheng et al., 2024). Another significant aspect is the age bracket of the participants, as research has demonstrated that social interactions with relatives and others improves mental-wellbeing among older adults, who normally have relatively fewer opportunities for socialization (Fernandez-Portero et al., 2023). Overall, engaging in dialogue and sharing their experiences enabled the participants to feel that their stress was alleviated, and they reported feeling more relaxed.

Past findings about DLGs have also found that they have contributed to improved mental well-being and its particular aspects (Fernández-Villardón et al., 2025; Morlà-Folch et al., 2022; Ruiz-Eugenio et al., 2023). In one study, DLGs were carried out with individuals with psychological disorders and reading a literary classic and discussing it over a year resulted in improved social relations and better emotional and social well-being, aspects of mental well-being (Zubiri-Esnaola et al., 2023). Additionally, DLGs were found to support mental health improvement among older women (Padrós-Cuxart et al., 2024). The present study expands the previous literature by studying the effect of DLGs within a South Asian context and with a specific group prone to higher levels of stress: carers of children with disabilities from a low- to middle-income background. The current and previous findings about DLGs associated with improved mental well-being can be understood within the context of socialization. DLGs provide a chance to build positive social relations, and social interaction is linked with an increase in well-being due to the perceived social support and trust that people build in speaking to others (Forsman et al., 2013).

### 3.3. Self-Development

Another theme identified from the interviews and focus groups was self-development in which different aspects of the individual self were seen as developing or being addressed. The participants reported experiencing a strengthening of their sense of self and self-identity. They engaged in reflective practices as they read the book relating the content to their lives and while engaging in dialogue when they listened to other participants sharing their parenting experiences. They would reflect on their own practices, evaluating and thinking about them. Below are some examples of when participants spoke about reflection.

“In the book there were themes that were wide ranging and that we all could relate to—from politics, family situations, friendships, teacher and student relations. It feels like we have come full circle in talking about all these different aspects of life. It’s like we got a chance to reflect on our life. How we have been spending it, it’s like we get a chance to stand back and think what we have done so far.”

(P5)

“I think many of us had the chance to reflect on our relationship with our spouse when we talked about marriage and other relations. When people talked about problems they faced in their marriages, I also thought about it. We make some mistakes when we are young and now, we can understand them better.”

(P8)

The participants had the chance to step back and think deeply about their life and what they have learned, to evaluate their personal growth. They also looked forward to coming to the sessions as expressed by a mother:

“Since we normally only focus on our children, this was something for us parents and family members.”

(P8)

Therefore, while they reflected on their role as parents or grandparents during the activity, they also appreciated having something meaningful just for themselves, beyond their responsibilities to their children. In fact, they were able to think of their individual identity beyond a parent or grandparent, as they are often seen normally within the school or at home, as expressed by one of the participants:

“It gave us the feeling that “we are something” as in we exist besides our roles as parents, we have our identity as a person, which we often forget in our busy lives.”

(P12)

The topic of identity was discussed during one of the sessions in which one of the women spoke about how no one called her by her name but rather by her relation either to her children or husband. So, she started to tell people to address her by her name. Other women joined in to this conversation and shared similar experiences and realizations. This ties in with the findings on Roma people’s participation in DLGs, where they found empowerment and a sense of identity by joining the DLGs (López de Aguileta, 2021). This may be explained by previous research, for instance in Serradell’s (2015) study through a biographical analysis of a migrant Moroccan woman who took part in DLGs in Spain and influenced other women in the social transformation of her community, suggesting that when marginalized individuals witness someone similar to themselves taking initiative, that person becomes a role model, inspiring empowerment and boosting their confidence (Serradell, 2015).

Based on the observations, a higher engagement was seen over subsequent sessions. Those who were reluctant to speak up during the sessions initially were speaking more confidently during the next sessions. These observations were also supported by the interviews, such as the sentiments expressed by a mother in the following quotation:

“I got more strength as I have started coming here, as I listened to the other parents. When I see their examples of how they talk about their children and how they proudly promote their children here, it makes me more confident about Sana (daughter). I feel that I am not alone, or I am not doing a bad job.”

(P8)

So, the DLGs helped reinforce their confidence in their roles as parents or grandparents. This aligns with a framework that proposes that caregivers find meaning and build a sense of identity in providing care for their child with disabilities (Avieli et al., 2022). Many participants in our study spoke about their primary focus as staying strong and providing care for their children who need them the most. Further, prior research has also found that DLGs promote the development of self-confidence among individuals (Yeste et al., 2017; León-Jiménez, 2020; Morlà-Folch et al., 2022). This may be attributed to the inclusive and supportive environment created through dialogic interactions (Ruiz-Eugenio et al., 2023), where participants feel heard, valued, and empowered to share their thoughts and experiences.

### 3.4. Social–Emotional Experiences and Well-Being

The results show that the participants experienced many emotional experiences and states of well-being as a result of the social interactions they had during the activity. Most agreed that before the DLGs they knew the other parents for quite a while but not at a deeper level and with limited interactions. Also, being at home, especially for mothers, it can be isolating not having someone of the same age to speak to. This is reflected in the words of these participants:

“I think that the gatherings have had a good impact because the parents get a chance to discuss their worries and family problems. I think that all people should have the chance to share their problems with other people. We cannot solve our problems in isolation.”

(P10)

“When we talked to other parents and got to know their challenges, that they are the same as ours, it gave us, as parents, more confidence. Sometimes we feel very alone with our problems in having a child with disabilities for example within our family.”

(P5)

Therefore, being involved in these sessions helps to reduce the loneliness that stay-at-home parents or caretakers may face due to not having interactions with other adults. Within a stressful lifestyle, such social interaction is likely to play a positive role as social support has been shown to decrease distress (Sánchez-Moreno et al., 2014). Further, as they engaged in positive social interactions and spoke about their common concerns, they found comfort in being able to express them. These conversations were quite emotional in which they expressed concerns about the future of their children with disabilities. As a grandmother recalled this conversation, she remarked,

“We got a sense of hope for our children.”

(P5)

In a similar vein, another mother said the following regarding the DLGs:

“Together we have examined different relationships, through this experience and talking with each other we got hope, happiness and we felt refreshed.”

(P11)

The participants expressed experiencing positive emotional states and improved well-being. This expression of emotions, especially during heavily charged emotional dialogue during one of the sessions, contributed to emotional resilience. The changes within self-identity explored earlier act as a precursor for developing greater emotional resilience; specifically, when there is a strong sense of self, emotionality is better expressed (Rajan-Rankin, 2014). Although occurring concurrently, the results demonstrate the strengthening of self and emotionality within the participants.

Another aspect related to the emotional well-being dimension is the distress experienced by parents of children with disabilities, who often lack formal support and are left to navigate their financial, medical, emotional, and other challenges on their own (Lakhani et al., 2024). One study highlights that, without reliable long-term support for their children, these parents are consistently anxious about who will care for their children after their passing (Rizvi Jafree & Burhan, 2020). In our study, parents expressed similar concerns, particularly the fear that their children might be vulnerable to abuse in their absence. Amid such emotionally taxing circumstances, they found comfort in attending the DLGs, where they experienced a sense of support, as expressed here:

“I felt very good coming here. Sometimes when I am sitting at home, I feel depressed. When I would come here, I used to feel more happy.”

(P12)

Overall, these findings are consistent with previous studies that found that DLGs helped improve social relations (Aubert et al., 2016; López de Aguileta, 2021; Padrós & Flecha, 2014; Serradell, 2015) but add to these findings by highlighting the emotional states experienced during these sessions. These emotional experiences may be attributed to the context in which the DLGs were conducted: a lower-income special education setting. As explored in previous studies, DLGs involve different community members who come from diverse backgrounds, but over the course of their interactions, they build deep bonds of friendship as they realize their shared experiences and values (Padrós & Flecha, 2014). In fact, a literature review revealed that one of the essential components of social cohesion is an orientation towards common good (Schiefer & Van der Noll, 2017). This concept helps explain why, in both the present study and previous findings, DLGs appear to bring people together. Participants recognize that they share common values, challenges, and goals, which creates a sense of solidarity and mutual support. As they engage in these shared dialogues, they develop a collective understanding that strengthens their emotional bonds and contributes to a more cohesive social environment.

### 3.5. Limitations and Future Research

The time period of this study during which the DLGs were conducted was two months. It may be useful to carry out DLGs for a longer period in a similar context which may reveal more, such as changes that may appear in the long term. The DLGs were discussed with the participants and school staff as an activity that can be held regularly, after the completion of the project. The sustainability of the social benefits of DLGs largely depends on the collective commitment of participants and school staff to carry on the activity and the opportunities they have to conduct it. In this particular case, a particular long-term impact that is likely to prevail is the social cohesion among this group of family members. After knowing each other at a deeper level and connecting over shared experiences, they may maintain their friendships. This is the reason that a long-term follow-up would be beneficial for future studies. As this study explored two novel dimensions with reference to DLGs, Pakistani culture and carers of children with disabilities, further investigations would expand the results. Further, the impacts of DLGs on carers of children with disabilities within Western culture may be explored to examine if there is a difference in the findings when the context and situation of carers is different.

## 4. Conclusions

This study highlights the significant benefits of dialogic literary gatherings (DLGs) in promoting cognitive development, mental well-being, self-development, emotional experiences, and increased socialization among middle-aged to older carers of children with disabilities. Through engaging in meaningful dialogue, participants demonstrated enhanced cognitive skills, such as critical thinking and reflection, while also experiencing improvements in their mental well-being. DLGs provide a safe space for individuals, particularly parents of children with disabilities, to share their challenges, alleviating feelings of isolation and distress. The process of self-expression and mutual support within these gatherings encouraged personal growth and development, reinforcing the participants’ sense of identity and confidence. Furthermore, the shared emotional experiences created strong social bonds, enhancing socialization and cultivating a sense of belonging and community. Overall, this study emphasizes the power of dialogic-based interventions in promoting holistic development, emotional resilience, and social cohesion among aging people who are at risk of social isolation and loneliness.

## Figures and Tables

**Table 1:** Sample of themes, subthemes, and units of meaning.

Themes	Subthemes	Units of Meaning
Cognitive engagement	Critical thinking	Compare, evaluate, criticize
Mental well-being	Reduced stress	Relaxed, calm, relieved, lighthearted
Self-development	Realization of self-identity	Confidence, worth, importance
Social–emotional experiences	Positive emotionality	Hope, grateful, happiness

## Data Availability

The dataset is available upon request from the authors.
